# Upregulation of long noncoding RNA SPRY4‐IT1 correlates with tumor progression and poor prognosis in cervical cancer

**DOI:** 10.1002/2211-5463.12102

**Published:** 2016-08-09

**Authors:** Yang Cao, Yinglei Liu, Xiaoyan Lu, Ying Wang, Haifeng Qiao, Manhua Liu

**Affiliations:** ^1^ Department of Obstetrics and GynecologyThe Second Affiliated Hospital of Nantong UniversityChina

**Keywords:** cervical cancer, lncRNA, prognosis, SPRY4‐IT1

## Abstract

The identification of cancer‐associated long noncoding RNA (lncRNA) is critical for us to understand cancer pathogenesis and development. The aim of this study was to evaluate the expression profile of the lncRNA SPRY4‐IT1 in cervical cancer and to identify its clinical significance in cancer progression. The expression levels of SPRY4‐IT1 in cervical cancer tissues were measured by quantitative real‐time PCR, and its correlation with overall survival of cervical cancer patients was analyzed statistically. Our results showed that the expression levels of SPRY4‐IT1 were higher in cervical cancer tissues than in adjacent normal tissues. Patients with higher SPRY4‐IT1 expression had advanced clinical characteristics and a shorter overall survival time than those with lower SPRY4‐IT1 expression. Moreover, multivariate analysis showed that relative SPRY4‐IT1 expression was an independent predictor of overall survival in patients with cervical cancer. In addition, the model we have established shows a good prediction of the probability of 5‐year overall survival of patients according to the *c*‐index and calibration curve. Collectively, our data suggest that lncRNA SPRY4‐IT1 may be a novel molecule involved in cervical cancer progression, which may be of use as both a potential predictor and therapeutic target.

**Abbreviations**
ccRCC: clear cell renal cell carcinoma
ESCC: esophageal squamous cell carcinoma
FIGO: Federation International of Gynecology and Obstetrics
lncRNA: long noncoding RNA
NSCLC: nonsmall‐cell lung cancer
qRT‐PCR: quantitative real‐time PCR
ROC: receiver operating characteristic

Cancer incidence and mortality have been increasing worldwide, and cancer has been a leading cause of death since 2010 1. Cervical cancer remains the fourth most common cancer in women and the leading gynecologic malignancy worldwide 2. Despite widespread implementation of Pap smear screening, cervical cancer remains a major public health problem 3. Therefore, major efforts have focused on the identification of a novel molecular biomarker for the early diagnosis or predicting the clinical prognosis in patients with cervical cancer.

Long noncoding RNA (lncRNA), which are longer than 200 nucleotides with no protein‐coding capability, provide key targets for cancer diagnoses and treatments due to their important functions in a variety of cellular processes including epigenetic changes, microRNA sequestering, and tumor suppressor activity 4. LncRNAs have been shown to be cell type‐ and temporal‐specific regulation of gene expression, and can regulate many cellular processes through multiple disparate mechanisms 5. SPRY4 intronic transcript 1 (SPRY4‐IT1: GenBank Accession ID AK024556), which is a 687 nucleotides unspliced, polyadenylated transcript derived from the second intron of the SPRY4 gene, is firstly identified in melanoma 6. The SPRY4‐IT1 has demonstrated its overexpression and amplification in multiple cancer types 7, 8, 9, 10, 11, 12, indicating that it may promote carcinogenesis. Moreover, SPRY4‐IT1 expression has been remarkably correlated with clinical characteristics such as risk, relapse, progression in esophageal squamous cell carcinoma (ESCC) 7, nonsmall‐cell lung cancer (NSCLC) 8, clear cell renal cell carcinoma (ccRCC) 10, gastric cancer 13, 14, bladder cancer 15, and melanoma 16.

Despite the wealth of knowledge regarding oncogenic effects of SPRY4‐IT1 in various cancers, very little is known about its precise prognostic significance. In fact, the handful of reports providing SPRY4‐IT1 prognostic data extremely suggest that it exhibits its prognostic value in several cancers. For example, patients with higher SPRY4‐IT1 expression have a poorer prognosis than those with lower SPRY4‐IT1 expression, and SPRY4‐IT1 is an independent prognostic indicator in patients with multiple cancers 7, 8, 10. However, its prognostic significance in cervical cancer remains unknown.

In this study, we aimed to investigate the expression of SPRY4‐IT1 in cervical cancer and to further explore its clinical significance in cervical cancer. We first assessed the expression profile of SPRY4‐IT1 in cervical cancer tissues by quantitative real‐time PCR (qRT‐PCR). Next, we evaluated its associations with clinical characteristics to identify its clinical significance in cervical cancer. In addition, we constructed a predictive model to predict clinical outcomes based on SPRY4‐IT1 expression in patients with cervical cancer.

## Materials and methods

### Ethics statement

This study protocol was approved by the Medical Ethics Committee of The Second Affiliated Hospital of Nantong University, and the written consent was obtained from each enrolled patient.

### Human tissues and serum samples

Tissue samples were obtained from a total of 100 patients with cervical cancer who underwent radical resections at The Second Affiliated Hospital of Nantong University. None of the patients had received chemotherapy or radiotherapy prior to surgery. Clinicopathological characteristics in this study are summarized in Table 1. Tumor tissues were obtained and stored immediately in liquid nitrogen after surgical resection. All samples were confirmed by a senior pathologist and were staged according to the Federation International of Gynecology and Obstetrics (FIGO) staging system for cervical cancer. Each patient was regularly followed up with a median follow‐up period of 53 months (range: 3–60 months).

**Table 1:** Correlation of SPRY4‐IT1 expression with clinical characteristics in cervical cancer

Factors	Tumor low expression (*n* = 54) *N* (%)	Tumor high expression (*n* = 46) *N* (%)	*P*
Age			
< 45 years	31 (57.4)	18 (39.1)	0.068
≥ 45 years	23 (42.6)	28 (60.9)	
Histology			
Squamous	42 (77.8)	36 (78.3)	0.954
Adenocarcinoma	12 (22.2)	10 (21.7)	
Tumor size (cm)			
< 4	43 (79.6)	21 (45.7)	< 0.001
≥ 4	11 (20.4)	25 (54.3)	
FIGO stage			
IB	37 (68.5)	18 (39.1)	< 0.001
IIA	14 (25.9)	13 (28.3)	
IIB	3 (5.6)	15 (32.6)	
Differentiation			
Well/moderate	35 (64.8)	38 (82.6)	0.046
Poor	19 (35.2)	8 (17.4)	
SCC‐Ag (μg·L^−1^)			
< 4	40 (74.1)	24 (52.2)	0.023
≥ 4	14 (25.9)	22 (47.8)	
Lymph node metastasis			
Negative	45 (83.3)	19 (41.3)	< 0.001
Positive	9 (16.7)	27 (58.7)	

Pearson chi‐square.

### RNA extraction and cDNA synthesis

Total RNA was extracted from tissue samples using the TRIzol reagent (Invitrogen, Carlsbad, CA, USA) according to the manufacturer instruction and stored at −80 °C. RNA concentration and purity were further measured on a NanoDrop spectrophotometer (Thermo Scientific, Waltham, MA, USA). The extracted RNA was used to perform the next experiments if its OD260/280 ranged from 1.8 to 2.0, and cDNA synthesis was obtained from 1 μg RNA template. Synthesis of cDNA was performed using PrimeScript RT reagent Kit with gDNA Eraser (Takara, Dalian, China) and stored at −20 °C until SPRY4‐IT1 expression analysis.

### Quantitative real‐time PCR

The expression levels of SPRY4‐IT1 were determined using SYBR Premix Ex Tag^™^ II (Takara) following the manufacturer recommendation by ABI 7500 System (Applied Biosystems, Foster City, CA, USA). The PCR reaction was performed in a volume of 30 μL containing 30 ng cDNA for each well. PCR cycling process was set as follows: initiate hold at 95 °C for 10 min, followed by 45 amplification cycles of melting at 95 °C for 15 s, annealing and extension at 60 °C.

For the normalization of tissue data, gene expression was normalized to the respective GAPDH expression level. The primer sequences of SPRY4‐IT1 were as follows: SPRY4‐IT1: forward: 5′‐ATCCGAAGCGCAGACACAATTCA‐3′; reverse: 5′‐CCTCGATGTAGTCTATGTCATAGGA‐3′; GAPDH: forward: 5′‐TGTGGGCATCAATGGATTTGG‐3′, reverse: 5′‐ACACCATGTATTCCGGGTCAAT‐3′. The relative expression was present as fold changes by the comparative *C*_t_ (ΔΔ*C*_t_) method.

### Statistical analysis

All statistical analyses were conducted using spss version 20.0 software (IBM, San Jose, CA, USA). Statistical significance was evaluated by a chi‐square test or Wilcoxon signed‐rank test as appropriate. Data were presented as mean ± SD or number (percentage) if necessary. Receiver operating characteristic (ROC) curve analysis was used to determine the optimal cutoff value of SPRY4‐IT1 in tumor/nontumor. Survival curves and log‐rank test were used to analyze patients' survival. Univariate and multivariate Cox regression analyses were employed to assess survival data. A predictive model was constructed based on significant variables in univariate analysis by r version 3.2.2 software (Institute for Statistics and Mathematics, Sydney, Austria), and Harrell's concordance index (*c*‐index) assessed its predictive efficiency. Values of *P* less than 0.05 were considered statistically significant.

## Results

### Upregulation of SPRY4‐IT1 in cervical cancer tissues

**Figure 1:**
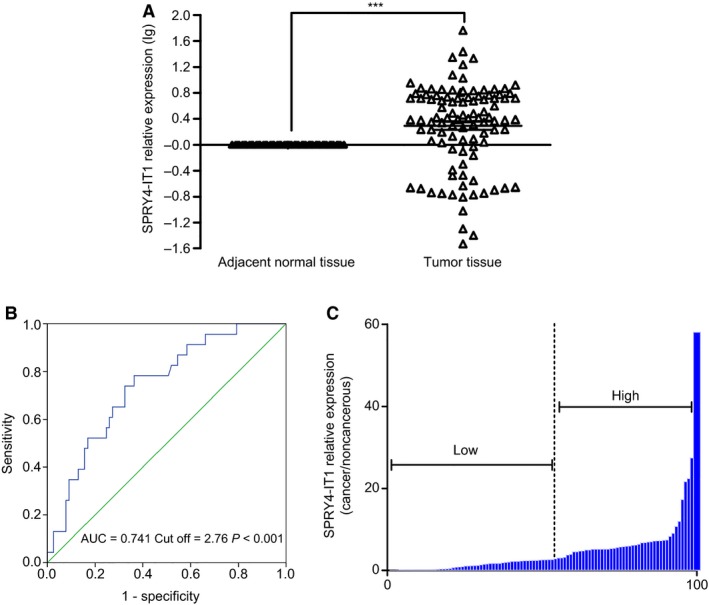
Relative SPRY4‐IT1 expression and its clinical significance in patients with cervical cancer. (A) SPRY4‐IT expression in cervical cancer tissues in comparison with adjacent normal tissues. The levels of SPRY4‐IT were measured by qRT‐PCR and normalized to GAPDH expression. (B) Receiver operating characteristic curve analysis was used to determine the optimal cutoff value of SPRY4‐IT1 in tumor/nontumor. (C) A total of 100 patients with cervical cancer were classified into a high‐SPRY4‐IT1 group (*n* = 46) and low‐SPRY4‐IT1 group (*n* = 54). ***P < 0.001.

Relative expression levels of SPRY4‐IT1 were determined by qRT‐PCR in a total of 100 patients with cervical cancer. Expression levels were normalized to 0 (log scale) in adjacent normal tissues, and SPRY4‐IT1 expression was remarkably increased in cervical cancer tissues compared to adjacent normal tissues (*P* < 0.001, Fig. 1A). ROC curve analysis shows that SPRY4‐IT1 expression is a good candidate to discriminate tumor tissues from normal tissues (sensitivity: 78.3%, specificity: 63.6%). Furthermore, the optimal cutoff value of SPRY4‐IT1 (2.76‐fold) in cancer/noncancer was determined by the largest Youden's index (0.419; sum of sensitivity and specificity − 1). Area under ROC curve (AUC) is 0.741 (95%CI: 0.632–0.849, *P* < 0.001; Fig. 1B). Then, patients with cervical cancer were classified into two groups based on the optimal cutoff value of relative expression by ROC curve analysis (Fig. 1C).

### Correlations between the expression of SPRY4‐IT1 and clinical characteristics in cervical cancer

To determine its clinical relevance in cervical cancer, we examined correlations between SPRY4‐IT1 expression and clinicopathlogical factors such as age, histology, tumor size, FIGO stage, tumor differentiation, SCC‐Ag, Lymph node metastasis. As shown in Table 1, upregulation of SPRY4‐IT1 was markedly correlated with tumor size, FIGO stage, SCC‐Ag, and lymph node metastasis (*P* < 0.05), but not correlated with patient's age and histology (*P* > 0.05). In addition, a borderline significance was observed between tumor differentiation and SPRY4‐IT1 expression (*P* = 0.046). Taken together, these findings suggest that upregulated SPRY4‐IT1 expression was correlated with the development and progression of cervical cancer.

### High SPRY4‐IT1 expression predicts poor prognosis in patients with cervical cancer

**Figure 2:**
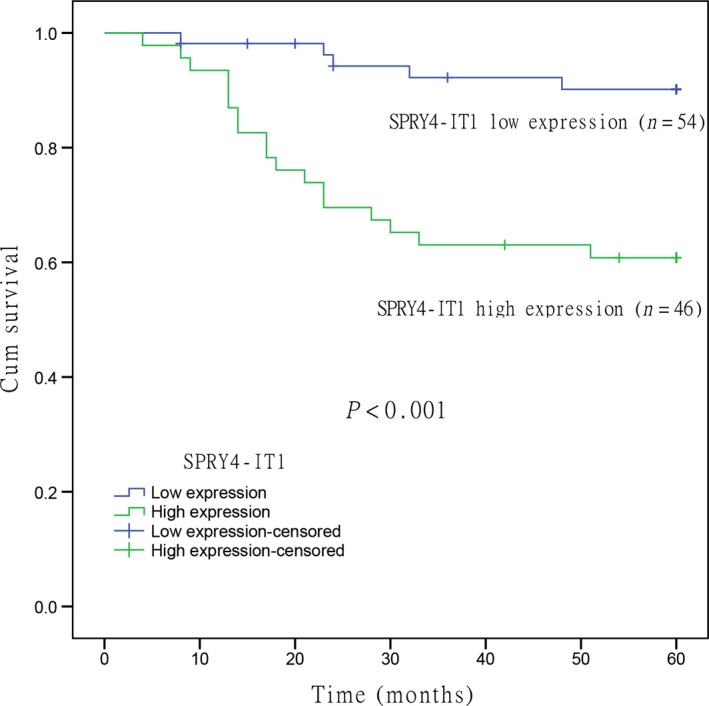
Kaplan–Meier curves for overall survival in patients with cervical cancer according to SPRY4‐IT1 expression. Patients with high SPRY4‐IT1 expression had significantly poorer overall survival than those with low SPRY4‐IT1 expression (*P* < 0.001, log‐rank test).

To further understand the clinical significance of SPRY4‐IT1 in cervical cancer, we first analyzed its survival data by Kaplan–Meier analysis. The results showed that cervical cancer patients with high SPRY4‐IT1 expression had significantly shorter overall survival time than those with low SPRY4‐IT1 expression (*P* < 0.001, Fig. 2). Univariate analysis suggested that SPRY4‐IT1 expression, tumor size, FIGO stage, SCC‐Ag, and lymph node status were significantly associated with worse overall survival in patients with cervical cancer (*P* < 0.05). Furthermore, relative SPRY4‐IT1 expression was an independent prognostic factor for overall survival of patients with cervical cancer in multivariate analysis (Table 2). These results revealed that SPRY4‐IT1 expression could serve as a potential independent prognostic factor in patients with cervical cancer.

**Table 2:** Univariate and multivariate analysis for overall survival in patients with cervical cancer

Risk factors	Category	Univariate analysis		Multivariate analysis	
		HR (95% CI)	*P*	HR (95% CI)	*P*
SPRY4‐IT1 in tumor	High (*n* = 46)/Low (*n* = 54)	4.95 (1.84–13.35)	0.002	3.87 (1.38–10.83)	0.010
Age (years)	≥ 45 (*n* = 51)/< 45 (*n* = 49)	1.21 (0.53–2.76)	0.648		
Histology	Adenocarcinoma (*n* = 22)/Squamous (*n* = 78)	1.00 (0.37–2.70)	0.996		
Tumor size (cm)	≥ 4 (*n* = 36)/< 4 (*n* = 64)	3.32 (1.43–7.67)	0.005	2.26 (0.95–5.40)	0.066
FIGO stage	IIA–IIB (*n* = 45)/IB (*n* = 55)	2.54 (1.08–6.00)	0.033	1.14 (0.35–3.66)	0.830
Differentiation	Poor (*n* = 27)/Well/moderate (*n* = 73)	0.90 (0.35–2.27)	0.815		
SCC‐Ag (μg?L^‐1^ )	≥ 4 (*n* = 36)/< 4 (*n* = 64)	3.32 (1.43–7.67)	0.005	1.22 (0.53–2.82)	0.639
Lymph node metastasis	Positive (*n* = 36)/Negative (*n* = 64)	3.19 (1.38–7.38)	0.007	1.74 (0.71–4.27)	0.226

### A predictive model for overall survival

**Figure 3:**
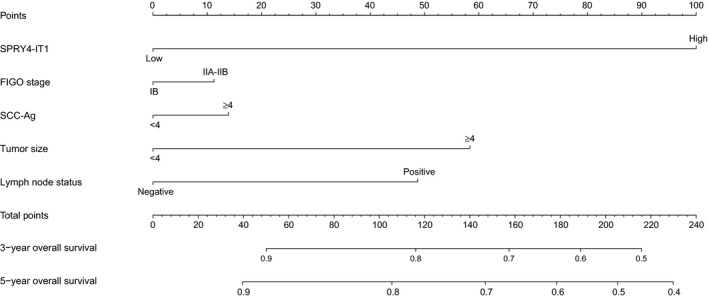
A predictive model according to clinical characteristics. Nomogram for survival of patients with cervical cancer.

**Figure 4:**
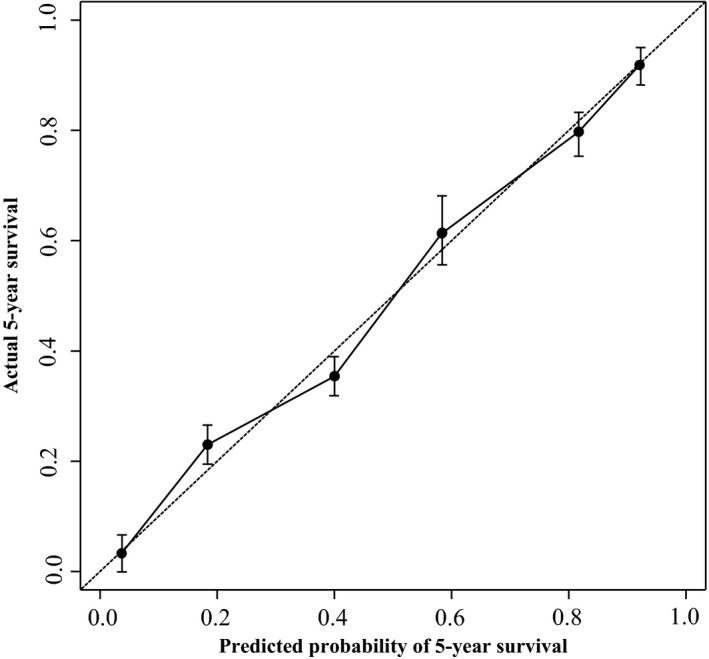
Calibration curve for 5‐year survival in cervical cancer patients. The dashed line shows an ideal nomogram, and the solid line refers to performance of the actual nomogram.

To precisely predict clinical prognosis of patients with cervical cancer, a prognostic nomogram was established using the significant factors identified in univariate analysis (Table 2). This model was used by summing the points identified on the top scale for each factor. Then, these total point scores were identified on the total points scale to observe the probability of 3‐ and 5‐year overall survival (Fig. 3). The *c*‐index for the model was 0.763 according to the fitted multivariable Cox regression analysis on the 100 patients. The calibration curve was used to determine how the predictions from the nomogram compared to the actual outcomes for the 100 patients. The dashed line presented the performance of an ideal nomogram, in which the predicted observations perfectly matched with the actual observations (Fig. 4).

## Discussion

Cervical cancer is one of the leading causes of death from gynecologic malignancies, so it is urgent for us to seek new potential biomarkers for its diagnosis, prognosis, and therapy to improve clinical strategies of cervical cancer. Recently, numerous lncRNAs have been characterized and reported to play an important role in cancer pathogenesis, suggesting that they may be involved in disease biology 17. Mounting evidence revealed that dysregulation of lncRNAs causes a range of biological functions leading a progressive and unlimited tumor growth 18. LncRNAs have been used to develop as biomarkers and prognostic factors 19. SPRY4‐IT1 is derived from an intron of the SPRY4 gene, which was originally associated with melanoma 6. Moreover, SPRY4‐IT1 has been discovered as a prognostic biomarker for several cancers. In this study, we sought to identify the expression profile of SPRY4‐IT1 in cervical cancer and further know its prognostic value in patients with cervical cancer.

We observed an increased expression of SPRY4‐IT1 in cervical cancer tissues compared with that in corresponding normal tissues. Besides, we showed that increased SPRY4‐IT1 expression was correlated with tumor size, FIGO stage, SCC‐Ag, and lymph node metastasis in cervical cancer. It is more important that we also proved that SPRY4‐IT1 expression was an independent predictor for overall survival. Our constructed model was used to precisely predict clinical prognosis of patients with cervical cancer. These results suggest that lncRNA SPRY4‐IT1 plays a direct role in the regulation of cervical cancer progression and may serve as a novel biomarker for cervical cancer.

SPRY4‐IT1 is transcribed from an intron of the SPRY4 gene and has several long hairpins in secondary structure. Knockdown of SPRY4‐IT1 contributed to the suppression of biologic functions in melanoma cells 6. Furthermore, Liu *et al*. 16 has demonstrated that lncRNA SPRY4‐IT1 was increased in the plasma of melanoma patients compared to that in healthy controls, and served as an independent predictor for overall survival. In addition, SPRY4‐IT1 was also upregulated in esophageal squamous cell carcinoma 7, prostate cancer 20, and gastric cancer 14. Although the molecular mechanism by which SPRY4‐IT1 contributes to the development and progression of cervical cancer is not further investigated in the present study, previous investigations have provided several clues for us. Xie *et al*. 14 revealed that SPRY4‐IT1 promoted gastric cancer cells growth through regulating epithelial‐mesenchymal transition (EMT) process. Shi *et al*. 12 showed that SPRY4‐IT1 contributed to the proliferation of breast cancer cells by upregulation of ZNF703 expression. Mazar *et al*. 11 indicated that knockdown of SPRY4‐IT1 in melanoma cells could increase acyl carnitine, fatty acyl chains, and triacylglycerol to induce apoptosis. Collectively, SPRY4‐IT may contribute to the progression and development of cancer via many types of approaches.

A good predictive model can help physicians to discriminate high‐risk patients to choose an appropriate therapy for patient. The nomogram has been developed to help physicians predict clinical prognosis in patients with various types of cancers 21. The FIGO stage is often used to predict clinical outcome according to risk of disease development and death. In this study, we also attempted to establish a predict model to predict the probability of 3‐ or 5‐year overall survival for patients with cervical cancer based on SPRY4‐IT1 and the significant variables in univariate analysis. The nomogram was conducted well in predicting the clinical outcome of patients with cervical cancer according to the *c*‐index and calibration curve. This model aims to calculate some of the heterogeneity in the same FIGO stage and provide a precise strategy of therapy.

In conclusion, we have demonstrated that SPRY4‐IT1 is upregulated in patients with cervical cancer, and correlated with tumor progression. These findings indicate that SPRY4‐IT is an important molecular biomarker for predicting prognosis and a potential target for cervical cancer therapy.

## Author contributions

YC and MHL conceived and designed the project, YC, XYL, and YW acquired the data, YC and HFQ analyzed and interpreted the data, YC and MHL wrote the paper.

## References

[1] Siegel RL , Miller KD and Jemal A (2016) Cancer statistics, 2016. CA Cancer J Clin 66, 7–30. 26742998 10.3322/caac.21332

[2] Crosbie EJ , Einstein MH , Franceschi S and Kitchener HC (2013) Human papillomavirus and cervical cancer. Lancet 382, 889–899. 23618600 10.1016/S0140-6736(13)60022-7

[3] Kodama J , Seki N , Masahiro S , Kusumoto T , Nakamura K , Hongo A and Hiramatsu Y (2010) Prognostic factors in stage IB‐IIB cervical adenocarcinoma patients treated with radical hysterectomy and pelvic lymphadenectomy. J Surg Oncol 101, 413–417. 20127891 10.1002/jso.21499

[4] Quinn JJ and Chang HY (2016) Unique features of long non‐coding RNA biogenesis and function. Nat Rev Genet 17, 47–62. 26666209 10.1038/nrg.2015.10

[5] Prensner JR and Chinnaiyan AM (2011) The emergence of lncRNAs in cancer biology. Cancer Discov 1, 391–407. 22096659 10.1158/2159-8290.CD-11-0209 PMC3215093

[6] Khaitan D , Dinger ME , Mazar J , Crawford J , Smith MA , Mattick JS and Perera RJ (2011) The melanoma‐upregulated long noncoding RNA SPRY4‐IT1 modulates apoptosis and invasion. Cancer Res 71, 3852–3862. 21558391 10.1158/0008-5472.CAN-10-4460

[7] Xie HW , Wu QQ , Zhu B , Chen FJ , Ji L , Li SQ , Wang CM , Tong YS , Tuo L , Wu M *et al* (2014) Long noncoding RNA SPRY4‐IT1 is upregulated in esophageal squamous cell carcinoma and associated with poor prognosis. Tumour Biol 35, 7743–7754. 24810925 10.1007/s13277-014-2013-y

[8] Sun M , Liu XH , Lu KH , Nie FQ , Xia R , Kong R , Yang JS , Xu TP , Liu YW , Zou YF *et al* (2014) EZH2‐mediated epigenetic suppression of long noncoding RNA SPRY4‐IT1 promotes NSCLC cell proliferation and metastasis by affecting the epithelial‐mesenchymal transition. Cell Death Dis 5, e1298. 24967960 10.1038/cddis.2014.256 PMC4611729

[9] Lee B , Mazar J , Aftab MN , Qi F , Shelley J , Li JL , Govindarajan S , Valerio F , Rivera I , Thurn T *et al* (2014) Long noncoding RNAs as putative biomarkers for prostate cancer detection. J Mol Diagn 16, 615–626. 25307116 10.1016/j.jmoldx.2014.06.009 PMC4210464

[10] Zhang HM , Yang FQ , Yan Y , Che JP and Zheng JH (2014) High expression of long non‐coding RNA SPRY4‐IT1 predicts poor prognosis of clear cell renal cell carcinoma. Int J Clin Exp Pathol 7, 5801–5809. 25337221 PMC4203192

[11] Mazar J , Zhao W , Khalil AM , Lee B , Shelley J , Govindarajan SS , Yamamoto F , Ratnam M , Aftab MN , Collins S *et al* (2014) The functional characterization of long noncoding RNA SPRY4‐IT1 in human melanoma cells. Oncotarget 5, 8959–8969. 25344859 10.18632/oncotarget.1863 PMC4253410

[12] Shi Y , Li J , Liu Y , Ding J , Fan Y , Tian Y , Wang L , Lian Y , Wang K and Shu Y (2015) The long noncoding RNA SPRY4‐IT1 increases the proliferation of human breast cancer cells by upregulating ZNF703 expression. Mol Cancer 14, 51. 25742952 10.1186/s12943-015-0318-0 PMC4350857

[13] Peng W , Wu G , Fan H , Wu J and Feng J (2015) Long noncoding RNA SPRY4‐IT1 predicts poor patient prognosis and promotes tumorigenesis in gastric cancer. Tumour Biol 36, 6751–6758. 25835973 10.1007/s13277-015-3376-4

[14] Xie M , Nie FQ , Sun M , Xia R , Liu YW , Zhou P , De W and Liu XH (2015) Decreased long noncoding RNA SPRY4‐IT1 contributing to gastric cancer cell metastasis partly via affecting epithelial‐mesenchymal transition. J Transl Med 13, 250. 26238992 10.1186/s12967-015-0595-9 PMC4522960

[15] Zhao XL , Zhao ZH , Xu WC , Hou JQ and Du XY (2015) Increased expression of SPRY4‐IT1 predicts poor prognosis and promotes tumor growth and metastasis in bladder cancer. Int J Clin Exp Pathol 8, 1954–1960. 25973088 PMC4396312

[16] Liu T , Shen S‐K , Xiong J‐G , Xu Y , Zhang H‐Q , Liu H‐J and Lu Z‐G (2016) Clinical significance of long noncoding RNA SPRY4‐IT1 in melanoma patients. FEBS Open Bio 6, 147–154. 10.1002/2211-5463.12030 PMC4821346 27239436

[17] Tsai MC , Spitale RC and Chang HY (2011) Long intergenic noncoding RNAs: new links in cancer progression. Cancer Res 71, 3–7. 21199792 10.1158/0008-5472.CAN-10-2483 PMC3057914

[18] Maruyama R and Suzuki H (2012) Long noncoding RNA involvement in cancer. BMB Rep 45, 604–611. 23186998 10.5483/BMBRep.2012.45.11.227 PMC4133807

[19] Esteller M (2011) Non‐coding RNAs in human disease. Nat Rev Genet 12, 861–874. 22094949 10.1038/nrg3074

[20] Mouraviev V , Lee B , Patel V , Albala D , Johansen TE , Partin A , Ross A and Perera RJ (2016) Clinical prospects of long noncoding RNAs as novel biomarkers and therapeutic targets in prostate cancer. Prostate Cancer Prostatic Dis 19, 14–20. 26503110 10.1038/pcan.2015.48

[21] Wierda WG , O'Brien S , Wang X , Faderl S , Ferrajoli A , Do KA , Cortes J , Thomas D , Garcia‐Manero G , Koller C *et al* (2007) Prognostic nomogram and index for overall survival in previously untreated patients with chronic lymphocytic leukemia. Blood 109, 4679–4685. 17299097 10.1182/blood-2005-12-051458

